# District Nursing Committees: A Danger

**Published:** 1908-03-28

**Authors:** 


### Nursing Administration.

### DISTRICT NURSING COMMITTEES: A DANGER.

Can the public take too great an interest in nursing matters? It may seem that an emphatic negative ought to be returned to this query, for the growing importance of the nurse to the community at large calls for continual liberality on the part of private persons and municipal bodies, and without the stimulus of personal interest the pressing claims made by the nursing service on the purse can hardly be maintained. There is a sense, indeed, in which the wider the circle of persons engaged in the business of conducting the affairs of nursing societies the more healthy the society will be. Associations depending for support on the contributions of a few wealthy individuals are exposed to downfall should such persons leave their neighbourhood or die. A wide circle of subscribers, a wide circle of representatives of subscribers, are essential to the building-up of an association for providing nurses on a broad basis, for raising the funds and laying down the lines on which the work is to proceed. But it is when the actual working of the nursing society is in question that the query about public interest needs reconsidering. In providing the country, as it is hoped to do, with a supply of nurses it is sometimes forgotten that a very serious responsibility is incurred in regard to the privacy of the home. It may safely be said that prior to the introduction of the trained nurse no real insight was ever obtained into the homes of the poor either in town or country. District visitors in certain select cases, among "nice people," were admitted to a degree of confidence in the household affairs. But the intimate in-and-out knowledge which the district nurse now obtains as a matter of course in respect of her patients was denied to any outsiders. The gentry were kept at arms' length, and their innocent guesses at the tone of the cottage home, its emotions and its morals may be studied with amazement in the pages of the goody-goody literature prepared for the young generation by kind-hearted literary ladies. The poor have a right to the privacy of their homes, to their own traditions and makeshifts, to their own ways of showing joy and sorrow, and to their own standard of taste and manners, so carefully kept in the background lest those of higher rank should scoff. Are we going to change all this by the advent of the district nurse, and expose all the little secrets of the cottage to the critical eye of leisured ladies in villages and country towns? The procedure adopted in the majority of nursing societies is for the nurse to bring her books once a week to the house of a committee lady, where one or two other persons deeply interested in nursing are assembled. Here cases are discreetly talked over under the name of business; the nurse relates her most moving experiences during the past week, states her difficulties with patients or, possibly, with the doctor, receives encouragement and advice, and departs leaving a hundred small items of village gossip to be discussed by the sympathising ladies, and, possibly, for conversation is hard to find in villages, to be imparted in strict confidence to friends equally "interested in nursing." This is no forced picture of what takes place in countless districts where the nurse "works under a committee." It is altogether bad. The managing committee, on which doctors practising in the area ought to be appointed ex officio, should not consist in addition, of more members than the secretary, the treasurer, and perhaps the president of the society; and of these one only ought to be appointed to consult with the nurse over any case in which she may require advice and to check the books. That the nurse should have one person of her own sex to whom she can refer confidentially in perplexities and on whose support she can rely may be considered a necessity. Beyond this one person of tried discretion no single detail of the work accomplished should be allowed to stray. The relations existing between the doctor and nurse are peculiar, and much misunderstood by the laity. The formality maintained by mutual consent between them, the strict code of etiquette which should guide the nurse in her reception and carrying out of the doctor's orders, the curt mode of conveying wishes acquired in the hurried hospital atmosphere by the doctor and seldom lost, all this is entirely misunderstood by kind and sympathising ladies who listen to what they can only half grasp. Thus friction is often quite innocently fostered between the doctor and his nurse, and, what is more serious, his private patients are placed in a position to pronounce on the conduct of his cases and take sides for or against his methods. The committee of management, with its one appointed adviser for the nurse, would in our judgment do wisely to place the appointment and dismissal of the nurse largely in the power of the medical men working in the district. Do what they will the committee are altogether in the hands of the doctors in respect of the successful working of the nursing service. If the doctor does not like the nurse, or considers her disloyal, he will not send for her to his cases. She will find herself reduced to looking up such mild cases as do not need medical advice, acting to some extent in the neighbourhood the part often played in past days by the unqualified assistant. She may bind up chilblains, bandage bad legs, and advise upon incipient colds. If she is to be of any real advantage to the neighbourhood she must work under the doctor. But the doctor will much prefer to employ one whom he has himself selected, acting in conjunction, it may be, with his treasurer and secretary, but so that his judgment for or against the candidate is recognised as a strong factor in the choice. In country districts the best results can only be attained where the doctor practically acts as medical director to the nurse, and where the interest of those who love detail not wisely but too well is kept in abeyance. For the sake of doctor, nurse, and patients the less nursing matters are discussed the better, and carelessness in this respect acts as a factor in retarding the further development of district nursing throughout the country.

